# Draft Genome Sequence of Bacillus safensis RP10, Isolated from Soil in the Atacama Desert, Chile

**DOI:** 10.1128/MRA.01150-19

**Published:** 2019-10-31

**Authors:** Erwin Strahsburger, Felipe Zapata, Inti Pedroso, Derie Fuentes, Paz Tapia, Raul Ponce, Michael McClelland, Jorge Valdes

**Affiliations:** a Doctoral Program in Agronomy in Desert and Arid Zones, Molecular Biotechnology Laboratory, Faculty of Renewal Natural Resources, Universidad Arturo Prat, Iquique, Chile; b Bio-Computing and Applied Genetics Division, Center for Systems Biotechnology, Fraunhofer Chile Research Foundation, Santiago, Chile; c Department of Microbiology & Molecular Genetics, University of California, Irvine, Irvine, California, USA; Queens College

## Abstract

Genome analysis of Bacillus safensis RP10, a strain from the soil of Atacama Desert in northern Chile, reflects a bacterium adapted to live in soil containing high levels of heavy metals, high salt conditions, and low carbon and energy sources.

## ANNOUNCEMENT

The Atacama Desert of Chile is the driest desert in the world (1, 2) and has soils naturally enriched in heavy metals and salts, which affects local agricultural activities (3). These soils also harbor species of the genus *Bacillus* (4, 5). This genus is adapted to live in many different environments (6, 7), and it has been used for biocontrol of agricultural plagues (8), for phytoremediation (9, 10), and as a probiotic (11). With the aim of understanding how *Bacillus* species have adapted to conditions in the Atacama Desert, we isolated *Bacillus* strains by mixing 1 g of soil with 9 ml of NaCl 0.8% and incubating at 50°C for 1 h. The solution was decanted, and 1 ml of the supernatant was spread on Luria broth (LB) agar and incubated at 37°C for 4 days under aerobic conditions. One of the colonies recovered was named RP10, and its genome was sequenced. The genomic DNA was purified using a QuickExtract bacterial DNA extraction kit (Epibio), and the library was constructed using a Nextera XT kit (Illumina). Whole-genome shotgun sequencing was performed using 2 × 250- bp (paired-end) reads on an Illumina MiSeq platform. A total of 14.2 million reads was obtained. Reads were filtered for a Phred quality score of at least 20 and assembled using the A5 pipeline (2015 Linux, default parameters) (12). Open reading frame prediction and annotation were performed using Prokka software version 1.11 (13).

*Bacillus* species identification was achieved by using the JSpeciesWS server online with BLAST (14) average nucleotide identity (ANIb) and MUMmer average nucleotide identity (ANIm) analysis (15–17). The RP10 strain was identified as a member of the species Bacillus safensis, and the highest identities were with *B. safensis* FO-36b (16S rRNA, 99.2%; ANIb, 98.83%; ANIm, 99.07%) and *B. safensis* JPL-MERTA-8-2 (ANIb, 98.7%; ANIm, 99.00%). Both strains were isolated from clean rooms at the Jet Propulsion Laboratory at NASA (Pasadena, CA). (18–19). A genome comparison between these three strains by Mauve software (20) and the Rapid Annotation using Subsystem Technology (RAST)-National Microbial Pathogen Database Resource (NMPDR) server with SEED view indicated that the RP10 genome contained an island enriched in phage-related genes (60 kb), an arsenic resistance operon, and other uncharacterized metabolic operons not found in the other two genomes.

The draft genome of Bacillus safensis RP10 consists of 3,813,379 bp distributed in 102 contigs, with an average GC content of 41.7%. The draft genome comprises 75 tRNAs and 20 T box leader sequences that are probably involved in a riboswitch mechanism described for Gram-positive bacteria (21). The RAST-NMPDR server with SEED view indicates that this strain carries genes and operons for resistance to arsenic (*arsRRBC*), lead (*epsABCDEFFHIKLMN* and *cadA*), copper (*copZA*), and manganese (*mntP*, *mnH*, and *mntABC*) and an operon with potential antimicrobial activity and 34% identity with the bacteriocin operon AS-48 of Enterococcus faecalis. Finally, the genome encodes diverse capabilities for synthesizing several vitamins, siderophores, and aromatic compounds, including tryptophan (*trpEDCFBA*) and several multidrug efflux pumps, which are potentially associated with the ability to survive under saline soil conditions with a low content of energy and carbon sources.

### Data availability.

This whole-genome shotgun project has been deposited at DDBJ/ENA/GenBank under the accession number MKXN00000000 with contigs under accession numbers MKXN01000001 to MKXN01000102, and the raw reads are available in the SRA under the accession number PRJNA345377.

## References

[1] Olson DM, Dinerstein E 1998 The global 200: a representation approach to conserving the Earth’s most biologically valuable ecoregions. Conserv Biol 12:502–515. doi:10.1046/j.1523-1739.1998.012003502.x.

[2] Betancourt JL, Latorre C, Rech JA, Quade J, Rylander KA 2000 A 22,000-year record of monsoonal precipitation from northern Chile’s Atacama Desert. Science 289:1542–1546. doi:10.1126/science.289.5484.1542. 10968788

[3] De Gregori I, Fuentes E, Rojas M, Pinochet H, Potin-Gautier M 2003 Monitoring of copper, arsenic and antimony levels in agricultural soils impacted and non-impacted by mining activities, from three regions in Chile. J Environ Monit 5:287–295. doi:10.1039/b211469k. 12729270

[4] Crits-Christoph A, Robinson CK, Barnum T, Fricke WF, Davila AF, Jedynak B, McKay CP, DiRuggiero J 2013 Colonization patterns of soil microbial communities in the Atacama Desert. Microbiome 1:28. doi:10.1186/2049-2618-1-28. 24451153 PMC3971613

[5] Drees KP, Neilson JW, Betancourt JL, Quade J, Henderson DA, Pryor BM, Maier RM 2006 Bacterial community structure in the hyperarid core of the Atacama Desert, Chile. Appl Environ Microbiol 72:7902–7908. doi:10.1128/AEM.01305-06. 17028238 PMC1694221

[6] Nicholson WL, McCoy LE, Kerney KR, Ming DW, Golden DC, Schuerger AC 2012 Aqueous extracts of a Mars analogue regolith that mimics the Phoenix landing site do not inhibit spore germination or growth of model spacecraft contaminants *Bacillus subtilis* 168 and *Bacillus pumilus* SAFR-032. Icarus 220:904–910. doi:10.1016/j.icarus.2012.06.033.

[7] Cortesão M, Fuchs FM, Commichau FM, Eichenberger P, Schuerger AC, Nicholson WL, Setlow P, Moeller R 2019 *Bacillus subtilis* spore resistance to simulated Mars surface conditions. Front Microbiol 10:333. doi:10.3389/fmicb.2019.00333. 30863384 PMC6399134

[8] Bhattacharya S, Das A, Samadder S, Rajan SS 2016 Biosynthesis and characterization of a thermostable, alkali-tolerant chitinase from *Bacillus pumilus* JUBCH08 displaying antagonism against phytopathogenic *Fusarium oxysporum*. 3 Biotech 6:1–8. doi:10.1007/s13205-016-0406-x. PMC4781814 28330157

[9] Tiwari S, Sarangi BK, Thul ST 2016 Identification of arsenic resistant endophytic bacteria from *Pteris vittata* roots and characterization for arsenic remediation application. J Environ Manage 180:359–365. doi:10.1016/j.jenvman.2016.05.029. 27257820

[10] Wu T, Xu J, Liu J, Guo W, Li X, Xia J, Xie W, Yao Z, Zhang Y, Wang R 2019 Characterization and initial application of endophytic *Bacillus safensis* strain ZY for improving phytoremediation of oil-contaminated saline soils. Front Microbiol 10:991. doi:10.3389/fmicb.2019.00991. 31134029 PMC6515983

[11] Mu Y, Cong Y 2019 *Bacillus coagulans* and its applications in medicine. Benef Microbes 10:679–688. doi:10.3920/BM2019.0016. 31203635

[12] Tritt A, Eisen JA, Facciotti MT, Darling AE 2012 An integrated pipeline for *de novo* assembly of microbial genomes. PLoS One 7:e42304. doi:10.1371/journal.pone.0042304. 23028432 PMC3441570

[13] Seemann T 2014 Prokka: prokaryotic genome annotation system. Bioinformatics 30:2068–2069. doi:10.1093/bioinformatics/btu153. 24642063

[14] Altschul SF, Gish W, Miller W, Myers EW, Lipman DJ 1990 Basic Local Alignment Search Tool. J Mol Biol 215:403–410. doi:10.1016/S0022-2836(05)80360-2. 2231712

[15] Richter M, Rosselló-Móra R, Glöckner FO, Peplies J 2016 JSpeciesWS: a Web server for prokaryotic species circumscription based on pairwise genome comparison. Bioinformatics 32:929–931. doi:10.1093/bioinformatics/btv681. 26576653 PMC5939971

[16] Espariz M, Zuljan FA, Esteban L, Magni C 2016 Taxonomic identity resolution of highly phylogenetically related strains and selection of phylogenetic markers by using genome-scale methods: the *Bacillus pumilus* group case. PLoS One 11:e0163098. doi:10.1371/journal.pone.0163098. 27658251 PMC5033322

[17] Federhen S, Rossello-Mora R, Klenk HP, Tindall BJ, Konstantinidis KT, Whitman WB, Brown D, Labeda D, Ussery D, Garrity GM, Colwell RR, Hasan N, Graf J, Parte A, Yarza P, Goldberg B, Sichtig H, Karsch-Mizrachi I, Clark K, McVeigh R, Pruitt KD, Tatusova T, Falk R, Turner S, Madden T, Kitts P, Kimchi A, Klimke W, Agarwala R, DiCuccio M, Ostell J 2016 Meeting report: GenBank microbial genomic taxonomy workshop (12–13 May, 2015). Stand Genomic Sci 11:15.

[18] Coil DA, Benardini JN, Eisen JA 2015 Draft genome sequence of *Bacillus safensis* JPL-MERTA-8-2, isolated from a Mars-bound spacecraft. Genome Announc 3:e01360-15. doi:10.1128/genomeA.01360-15. 26586895 PMC4653797

[19] Satomi M, La Duc MT, Venkateswaran K 2006 *Bacillus safensis* sp. nov., isolated from spacecraft and assembly-facility surfaces. Int J Syst Evol Microbiol 56:1735–1740. doi:10.1099/ijs.0.64189-0. 16902000

[20] Darling ACE, Mau B, Blattner FR, Perna NT 2004 Mauve: multiple alignment of conserved genomic sequence with rearrangements. Genome Res 14:1394–1403. doi:10.1101/gr.2289704. 15231754 PMC442156

[21] Chopin A, Biaudet V, Ehrlich SD 1998 Analysis of the *Bacillus subtilis* genome sequence reveals nine new T-box leaders. Mol Microbiol 29:662–664. doi:10.1046/j.1365-2958.1998.00912.x. 9720882

